# Implementation of the infection prevention and control core components at the national level: a global situational analysis

**DOI:** 10.1016/j.jhin.2020.11.025

**Published:** 2021-02

**Authors:** E. Tartari, S. Tomczyk, D. Pires, B. Zayed, A.P. Coutinho Rehse, P. Kariyo, V. Stempliuk, W. Zingg, D. Pittet, B. Allegranzi

**Affiliations:** a Infection Prevention and Control Programme, Geneva University Hospitals, and Faculty of Medicine, Geneva, Switzerland; b Institute of Global Health, Faculty of Medicine, University of Geneva, Geneva, Switzerland; c Infection Prevention and Control Technical and Clinical Hub, Department of Integrated Health Services, World Health Organization (WHO), Geneva, Switzerland; d WHO Antimicrobial Resistance and Infection Prevention and Control Unit, Regional Office for the Eastern Mediterranean, Cairo, Egypt; e Health Emergencies Programme, WHO Regional Office for Europe, Copenhagen, Denmark; f Equipe d’Appui Interpays pour l’Afrique Centrale, WHO Country Office, Libreville, Gabon; g Pan American Health Organization Office for Jamaica, Bermuda and the Cayman Islands, Kingston, Jamaica

**Keywords:** Healthcare-associated infection, Infection prevention and control, Antimicrobial resistance, Core components, Implementation, World Health Organization

## Abstract

**Background:**

Strengthening infection prevention and control (IPC) is essential to combat healthcare-associated infections, antimicrobial resistance, and to prevent and respond to outbreaks.

**Aim:**

To assess national IPC programmes worldwide according to the World Health Organization (WHO) IPC core components.

**Methods:**

Between June 1^st^, 2017 and November 30^th^, 2018, a multi-country, cross-sectional study was conducted, based on semi-structured interviews with national IPC focal points of countries that pledged to the WHO ‘Clean Care is Safer Care’ challenge. Results and differences between regions and national income levels were summarized using descriptive statistics.

**Findings:**

Eighty-eight of 103 (85.4%) eligible countries participated; 22.7% were low-income, 19.3% lower-middle-income, 23.9% upper-middle-income, and 34.1% high-income economies. A national IPC programme existed in 62.5%, but only 26.1% had a dedicated budget. National guidelines were available in 67.0%, but only 36.4% and 21.6% of countries had an implementation strategy and evaluated compliance with guidelines, respectively. Undergraduate IPC curriculum and in-service and postgraduate IPC training were reported by 35.2%, 54.5%, and 42% of countries, respectively. Healthcare-associated infection surveillance was reported by 46.6% of countries, with significant differences ranging from 83.3% (high-income) to zero (low-income) (*P* < 0.001); monitoring and feedback of IPC indicators was reported by 65.9%. Only 12.5% of countries had all core components in place.

**Conclusion:**

Most countries have IPC programme and guidelines, but many less have invested adequate resources and translated them in implementation and monitoring, particularly in low-income countries. Leadership support at the national and global level is needed to achieve implementation of the core components in all countries.

## Introduction

Healthcare-associated infections (HCAIs) and antimicrobial resistance (AMR) significantly affect the quality and safety of healthcare delivery. Their impact on morbidity and mortality has been well described, including the economic burden on society [[1], [2], [3]]. The 2016–2017 point prevalence survey of the European Centre for Disease Prevention and Control reported that 8.9 million HCAIs occur every year in European acute and long-term care facilities [4]. Their previous estimates reported a total of 501 disability-adjusted life years lost per 100,000 population due to HCAIs, with more than 90,000 deaths per year [2]. In 2018, a point prevalence survey conducted in hospitals in the USA estimated that 3.2% of patients had one or more HCAIs [5]. In 2015, HCAI prevalence of 9% was estimated in South East Asia [6]. The World Health Organization (WHO) observed that HCAI prevalence was highest in low-/middle-income countries, ranging from 5.7% to 19.1% [1]. However, HCAIs are potentially avoidable if effective infection prevention and control (IPC) interventions are implemented, and reductions between 35% and 55% have been described [[7], [8], [9], [10], [11]].

The spread of infection in healthcare facilities is often at the origin of major outbreaks or determines their amplification. In an era when global public health emergencies and emerging AMR threaten major achievements in healthcare, strengthening IPC structure and organization at national level is key to ensure readiness to respond to outbreaks and to maintain and further improve safety in health care overall. Some studies have assessed IPC programmes nationwide or in multiple countries, but all were conducted at the facility level in high-income countries [[12], [13], [14], [15], [16]].

In 2016, WHO published evidence-based and expert consensus-based recommendations on effective IPC strategies summarized in eight core components, followed by the minimum requirements for their implementation in 2018 [17]. The aim was to support capacity-building to prevent HCAIs and AMR at both the national and facility levels, including epidemic events, and to work towards safe and resilient health systems [18,19]. While all eight core components address acute-care facilities, six are also relevant at the national level (Supplementary Table S1) [18]. This study was a global situational analysis conducted across all six WHO regions to assess the implementation level of the IPC core components at the national level.

## Methods

The study conduct and reporting is based on the Strengthening the Reporting of Observational Studies in Epidemiology (STROBE) guidelines [20].

### Study design and participants

Between June 2017 and November 2018, we conducted a multi-country, cross-sectional study based on semi-structured interviews with national focal points for IPC in ministries of health or other governmental organizations. For this study, we purposefully identified the 140 WHO Member States which had signed the First Global Patient Safety Challenge ‘Clean Care is Safer Care’ pledge to commit to actions to reduce HCAIs and improve patient safety (Supplementary Figure S1). Subsequently, we calculated a stratified sample size based on WHO region and World Bank country income levels to achieve representativeness of the final results [21,22]. The sample size calculation was based on the total of 140 ‘Clean Care is Safer Care’ pledge countries, 95% confidence intervals (CI), and the expected proportion of countries with an IPC programme, which was estimated to be ∼50% based on previous reports [23]. An ideal sample size of 103 countries was estimated, including the following proportions by WHO region: Western Pacific, 10/14; Eastern Mediterranean, 11/15; South East Asia, 4/6; Europe, 24/33; Africa, 28/38; and the Americas, 25/34. A convenience sample of national focal points to be interviewed were then identified through WHO and/or indicated by their country health authorities.

### Data sources and measurement

#### Survey instrument

The survey instrument was a questionnaire based on research literature on WHO IPC national core components published in 2016 and the study objectives [18,19]. The questionnaire was developed in collaboration with IPC international experts through a modified Delphi process [24]. Following the concept of the six national core components of IPC recommended by WHO, the survey structure was subdivided into six sections: (1) IPC programme; (2) IPC guidelines; (3) IPC education and training; (4) surveillance of HCAI and AMR; (5) multi-modal improvement strategies; and (6) monitoring, audit and feedback of IPC indicators. The final questionnaire included 30 items assessing implementation of IPC core components (Supplementary Questionnaire). The survey included dichotomous (yes/no) and closed-ended questions. Every answer to a question was allocated a numerical score. The survey instrument was translated into French, Spanish, and Russian from the original English version.

#### Pilot study

Before survey roll-out, a pilot study was performed in a convenience sample of experts from eight countries participating in a WHO international meeting to assess the questionnaire's validity. The pilot study was carried out to ensure comprehension and acceptability of the questions. The interviews of the pilot study were all done face-to-face and were in English only. Minor modifications were made on the basis of this pilot study.

#### Interview design

Data were collected through individual interviews from June 2017 to November 2018. The survey was administered over the telephone (*N* = 58) or face-to-face (*N* = 30) after verbal consent had been obtained. Priority was given to face-to-face interviews, if possible, during events such as international WHO meetings, congresses, or WHO country visits/missions. Telephone interviews were organized when face-to-face interviews were not possible. Invitations to participate in the study were sent via e-mail. Participation in this study was voluntary. All participants received information describing the aims and procedures of the study, the interview completion time (60–90 min), assurance of the confidentiality and anonymity of the survey responses and data reporting plans. Interviews were performed by trained study investigators (E. Tartari, S. Tomczyk, D. Pires, P. Kariyo, B. Zayed).

At least two email reminders were sent to the national focal points to encourage participation. With permission, all interviews were digitally audio-recorded and transcribed, and transcripts were reviewed for correctness. All data were entered into an Excel spreadsheet (Microsoft, Redmond, WA, USA) and double-checked for completeness by study investigators. Where necessary, checks to clarify responses were made by referring back to study participants and WHO regional focal points.

### Statistical analysis

Data were analysed using descriptive statistics; median (interquartile range (IQR)) or mean (95% confidence interval (CI)) were reported where applicable. Comparisons were made between the six WHO regions and World Bank income levels (high-income, upper-middle-income, lower-middle-income, and low-income) [22]. Data are reported in aggregate form by region and income level, without personal or country identifiers. Differences in categorical variables between groups were analysed using Fisher's exact test. Statistical significance was defined as two-sided *P* < 0.05. Statistical analyses were performed using R Studio 3.5.1 (R Foundation for Statistical Computing; 2017; https://www.r-project.org/).

### Ethical approval

The study was approved by the WHO ethics committee (ERC.0002994).

## Results

Eighty-eight of the 103 (85.4%) eligible countries participated in the study (Africa, 26/38, 68.4%; Eastern Mediterranean, 12/15, 80%; Europe, 20/30, 60.7%, the Americas, 21/34, 61.8%; South East Asia, 1/6, 16.7%; and the Western Pacific, 8/14, 57.1%) (Supplementary Figure S2). Country distribution by income levels was as follows: high-income, 34.1% (30/88); upper-middle-income, 23.9% (21/88); lower-middle-income, 19.3% (17/88), and low-income, 22.7% (20/88). Only 12.5% of countries reported to have all six key core components in place.

### Core component 1: IPC programme

**Figure 1:**
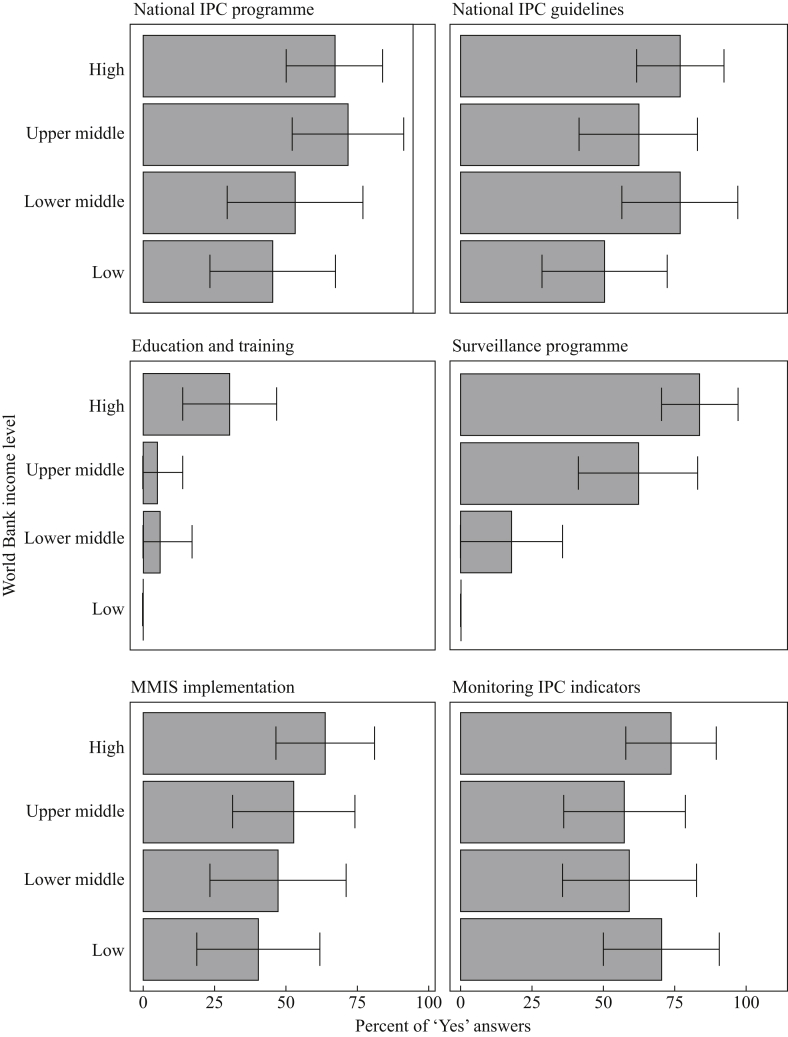
‘Clean Care is Safer Care’ participating countries situational analysis, 2017–2018 (*N* = 88): results of the six core components of infection prevention and control programmes stratified by the World Bank income level classification. Bars and whiskers indicate the overall percentage of ‘Yes’ responses with 95% confidence intervals. High-income (*N* = 30); upper-middle-income (*N* = 21); lower-middle-income (*N* = 17); low-income (*N* = 20). For Education and Training core component we calculated the percentage of countries that reported having all three types of IPC training (i.e. undergraduate, in-service, and postgraduate training). IPC, infection prevention and control; MMIS, multi-modal improvement strategy.

National IPC programmes for HCAI prevention with an appointed technical team or a focal person were available in 62.5% (55/88; 95% CI: 52.4–72.6) of countries, with variation across country income levels: from 70% (21/30; 95% CI: 53.6–86.4) in high-income to 45% (9/20; 95% CI: 23.2–66.8) in low-income countries (Figure 1). Disparities were observed across regions, with a lower frequency in Africa (46.2%; 12/26; 95% CI: 26.9–65.3) and the Eastern Mediterranean (58.3%; 7/12; 95% CI: 30.4–86.2) (*P* < 0.01) (Supplementary Figure S3). Only 26.1% (23/88; 95% CI: 16.9–35.3) linked the national programme with a dedicated budget for IPC activities. Whereas the majority of the countries (73; 83.0%; 95% CI: 75.1–90.8) had a national action plan to combat AMR, only 60.2% (53/88; 95% CI: 50.0–70.4) specifically included IPC in the AMR national action plan. Significant variations were observed between income levels, with a higher frequency of programmes in high- and upper-middle-income countries (*P* = 0.01).

### Core component 2: IPC guidelines

Approximately two-thirds (67%; 59/88; 95% CI: 57.2–76.8) of countries had national IPC guidelines available, with the lowest frequency reported in the Eastern Mediterranean and African regions (*P* = 0.04) (Supplementary Figure S2), as well as in low-income countries (*P* = 0.19) (Figure 1). Among countries with IPC guidelines, 36.4% (32/88; 95% CI: 26.3–46.5) had documents on implementation strategies. Furthermore, only 21.6% (19/88; 95% CI: 13.0–30.2) also monitored compliance with IPC practices recommended in the guidelines using process and outcome parameters (Table I). National guidelines for IPC measures, such as hand hygiene, standard and transmission-based precautions, were reported by most countries (89.8% (53/59; 95% CI: 82.1–97.5), 76.3% (45/59; 95% CI: 65.4–87.1) and 64.4% (38/59; 95% CI: 52.1–76.6), respectively). Specific guidelines targeting priority pathogens and infections recommended by WHO, such as meticillin-resistant *Staphylococcus aureus* (MRSA), multidrug-resistant Gram-negative organisms (i.e. carbapenem-resistant Enterobacterales (CRE), carbapenem-resistant *Pseudomonas aeruginosa* (CRPsA), carbapenem-resistant *Acinetobacter baumannii* (CRAB) and *Clostridioides difficile*) were reported by 52.5%, 37.3%, and 23.7% of countries, respectively.

**Table I:** Key elements of national IPC core components and a comparison according to the World Bank country income level classification

National IPC core components	All countries	Comparison between World Bank income levels				*P*-value
		High income	Upper-middle income	Lower-middle income	Low income	
Details of selected core components	*N* = 88	*N* = 30	*N* = 21	*N* = 17	*N* = 20	
Guideline implementation strategy	32 (36.4%)	17 (56.7%)	6 (28.6%)	5 (29.4%)	4 (20%)	0.04
Monitoring guideline compliance	19 (21.6%)	11 (36.7%)	5 (23.8%)	2 (11.8%)	1 (5%)	0.04
Master level training in IPC	16 (18.2%)	10 (33.3%)	5 (23.8%)	1 (5.9%)	0	<0.01
Doctoral training in IPC	8 (9.1%)	6 (20%)	2 (9.5%)	0	0	<0.05
National reference laboratory for HCAIs and AMR	65 (73.9%)	28 (93.3%)	16 (76.2%)	12 (70.6%)	9 (45%)	0.01
Understanding of MMIS	45 (51.1%)	18 (60%)	12 (57.1%)	8 (47%)	7 (35%)	0.33
Monitoring indicators	*N* = 58	*N* = 22	*N* = 12	*N* = 10	*N* = 14	
Hand hygiene compliance	29 (50%)	14 (63.6%)	6 (50%)	4 (40%)	5 (35.7%)	0.38
Alcohol-based hand-rub consumption	16 (27.6%)	9 (40.9%)	4 (33.3%)	1 (10%)	2 (14.3%)	0.19
WASH indicators	34 (58.6%)	3 (13.6%)	8 (66.7%)	9 (90%)	14 (100%)	<0.01
Antibiotic consumption	23 (39.7%)	13 (59.1%)	6 (50%)	2 (20%)	2 (14.3%)	0.025
Healthcare worker staffing levels	14 (24.1%)	6 (27.3%)	5 (41.7%)	2 (20%)	1 (7.1%)	0.22
Bed occupancy	21 (36.2%)	10 (45.5%)	7 (58.3%)	2 (20%)	2 (14.3%)	0.06
Other	7 (12.1%)	6 (27.3%)	0	1 (10%)	0	0.04
HCAI surveillance	*N* = 41	*N* = 25	*N* = 13	*N* = 3	*N* = 0	
CAUTI	28 (68.3%)	14 (56%)	13 (100%)	1 (33.3%)	0	<0.01
HAP/VAP	24 (58.5%)	13 (52%)	11 (84.6%)	0	0	<0.01
CLABSI	31 (75.6%)	17 (68%)	13 (100%)	1 (33.3%)	0	<0.01
SSI	34 (82.9%)	22 (88%)	11 (84.6%)	1 (33.3%)	0	0.14
AMR surveillance	*N* = 41	*N* = 25	*N* = 13	*N* = 3	*N* = 0	
*Clostridioides difficile* infection	21 (51.2%)	16 (64%)	4 (30.8%)	1 (33.3%)	0	0.14
MRSA	34 (82.9%)	21 (84%)	11 (84.6%)	2 (66.7%)	0	0.65
CRE, CRAB and/or CRPsA	28 (68.3%)	16 (64%)	11 (84.6%)	1 (33.3%)	0	0.16
ESBL-producing Enterobacterales	19 (46.3%)	12 (48%)	6 (46.2%)	1 (33.3%)	0	1

IPC, infection prevention and control; HCAI, healthcare-associated infection; AMR, antimicrobial resistance; MMIS, multi-modal improvement strategy; WASH, water sanitation and hygiene; CAUTI, catheter-associated urinary tract infection; HAP/VAP, healthcare/ventilator-associated pneumonia; CLABSI, central-line-associated bloodstream infection; SSI, surgical site infection; MRSA, meticillin-resistant *Staphylococcus aureus*; CRE, carbapenem-resistant Enterobacterales; CRAB, carbapenem-resistant *Acinetobacter baumannii*; CRPsA, carbapenem-resistant *Pseudomonas aeruginosa*.

### Core component 3: education and training in IPC

**Figure 2:**
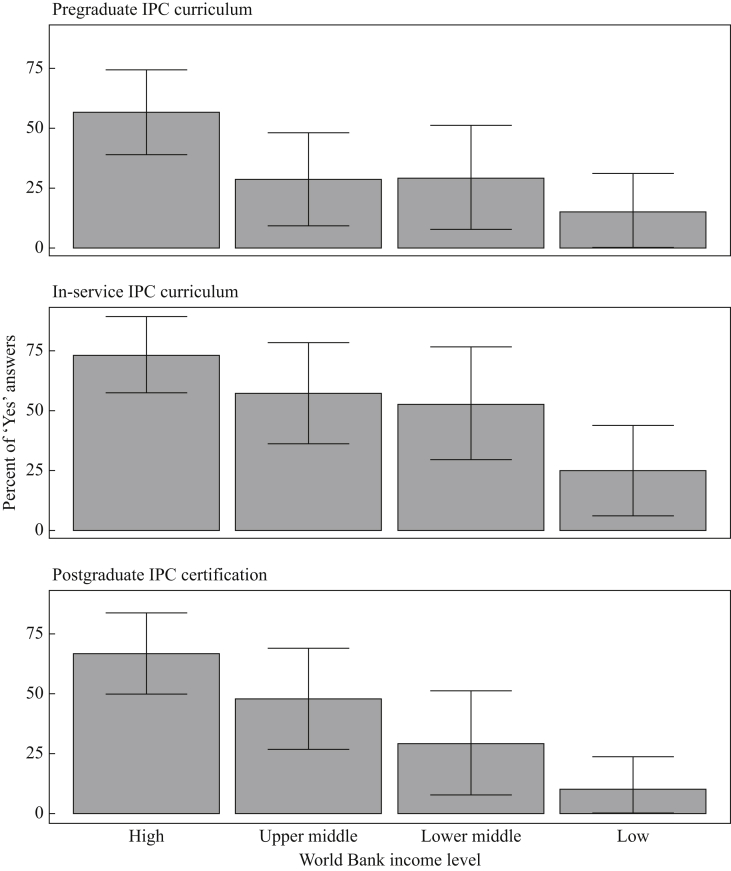
‘Clean Care is Safer Care’ participating countries situational analysis, 2017–2018 (*N* = 88): results of core component 3 (Education and Training) stratified by the World Bank income level classification. Bars and whiskers indicate the overall percentage of ‘Yes’ responses with 95% confidence intervals. High income (*N* = 30); upper-middle income (*N* = 21); lower middle-income (*N* = 17); low income (*N* = 20).

IPC education and training at undergraduate, in-service, and postgraduate levels were reported by 35.2% (95% CI: 25.2–45.2), 54.5% (95% CI: 44.1–64.9) and 42.1% (95% CI: 31.8–52.4) of countries, respectively, with significant differences across income levels (Figure 1, Figure 2) and regions (Supplementary Figures S3 and S4). Master and doctoral IPC programmes were available in 18.2% (16/88; 95% CI: 10.1–26.2) and 9.1% (8/88; 95% CI: 3.1–15.1) of countries, respectively (Table I). Training content was based on WHO guidelines (22/37, 59.5%; 95% CI: 43.6–75.2), US Centers for Disease Control and Prevention documents (18/37, 48.6%; 95% CI: 32.5–64.7), European Centre for Disease Prevention and Control, Training in Infection Control in Europe (TRICE) recommendations (11/37, 29.7%; 95% CI: 15–44.4), and the Association for Professionals in Infection Control and Epidemiology tools (8/37, 21.6%; 95% CI: 8.3–34.8).

### Core component 4: surveillance of outcome indicators

Most countries (73.9%, 65/88; 95% CI: 64.7–83.1) had a national AMR reference laboratory, with a significant variation across income levels (*P* < 0.01) (Table I). Less than half (41/88, 46.6%; 95% CI: 36.2–57.0) had established national surveillance networks on HCAI. Marked disparities were observed across regions with a lower frequency in Africa (3.8%, 1/26; 95% CI: 0–11.2) and the Eastern Mediterranean (25%, 3/12; 95% CI: 0.5–49.5) (*P* < 0.01) (Supplementary Figure S2). The extremes were 83.3% (25/30, 95% CI: 70–96.6) in high-income and zero in low-income countries (Figure 1). Surveillance most often addressed surgical site infection, followed by central-line-associated bloodstream infection, catheter-associated urinary tract infection, and healthcare/ventilator-associated pneumonia (Table I). Meticillin-resistant *Staphylococcus aureus* was the most common ‘alert’ organism, followed by carbapenem-resistant Enterobacterales, *Acinetobacter baumannii* and *Pseudomonas aeruginosa*, *Clostridioides difficile*, and extended-spectrum β-lactamase-producing Enterobacterales (Table I).

### Core component 5: multi-modal improvement strategies

More than half of the countries (58%, 51/88; 95% CI: 40.7–61.5) reported an understanding of multi-modal improvement strategies with no significant disparities (Table I). National IPC teams supported and co-ordinated multi-modal improvement strategies for the implementation of IPC interventions in 52.4% (47/88; 95% CI: 41.9–62.7), with variations across income levels (Figure 1) and regions (Supplementary Figure S2). The most frequently reported use of these strategies was mentioned in the context of hand hygiene improvement (51/51; 100%), followed by prevention programmes for surgical site infection (28/51, 54.9%; 95% CI: 41.2–68.5), central-line-associated bloodstream infection (26/51, 51%; 95% CI: 37.2–64.7), catheter-associated urinary tract infection (15/51, 29.4%; 95% CI: 16.9–41.9), and healthcare/ventilator-associated pneumonia (11/51, 21.6%; 95% CI: 10.2–32.8). Multi-modal strategies in the context of antimicrobial stewardship programmes were mentioned by 28 countries (28/51, 54.9%; 95% CI: 41.2–68.5).

### Core component 6: monitoring, audit and feedback of IPC indicators

IPC-related indicators (hand hygiene compliance, alcohol-based hand-rub consumption, those related to water and sanitation systems, antibiotic consumption, healthcare worker staffing levels, and bed occupancy) were monitored in 65.9% (58/88; 95% CI: 56.0–75.8) of countries (Table I), but poorly in low-income nations (Figure 1). Significant regional differences were observed, with the highest frequency observed in Europe, the Americas, and the Western Pacific (*P* = 0.01) (Supplementary Figure S2).

## Discussion

To our knowledge, this is the first global study providing a detailed situational analysis on the implementation of national IPC programmes across the six WHO regions and World Bank income levels. Other regular international assessments have addressed some IPC aspects, notably the annual AMR global survey to evaluate the AMR national action plans, but no detailed data on key elements of IPC programmes and their implementation have been available so far [25]. Our study, similar to the 2018 AMR global survey, showed an increase in the proportion of countries with a national IPC programme (62.5% and 88.6%, respectively) compared to the 2015 AMR survey (41%) [26,27]. This difference can be partly explained by the fact that the AMR global action plan was issued in 2015 and the WHO guidelines on IPC core components in 2016. Since then, many efforts have been made to support implementation by countries; for example, WHO developed guidance and toolkits that provide practical approaches for the implementation of the IPC core component and best practices based on evidence and country examples [28]. In addition, training packages have been produced and made available by WHO through both in-person and e-learning courses (https://ipc.ghelearning.org/courses; https://openwho.org/channels/ipc). Dissemination of these implementation resources has been achieved through the work of WHO regional and country offices and partners included in the WHO-led Global IPC Network (https://www.who.int/infection-prevention/about/GIPC_Network/en/).

Gaps in national IPC activities impact on a country's ability to meet the International Health Regulations, respond to highly infectious disease outbreaks, and combat AMR, thus impeding the achievement of the health-related United Nations Sustainable Development Goals, including quality care within universal health coverage [25,29].

Compared to self-assessment surveys, the major strength of our global survey methodology was the ability to discuss with national IPC focal points and to jointly agree on the most appropriate answers to the study questions in order to reflect countries' real situation with regards to IPC. This approach allowed us to gather many interesting details elaborated upon in the analysis and may also explain some differences in findings compared to other surveys with similar questions but using different methods. Only 12.5% of participating countries had elements of all six core components in place. Low adoption was identified, particularly for the core components on the surveillance of HCAI and the monitoring of IPC indicators in low-income countries. Although more WHO core components were in place in high-income countries, considerable gaps were still identified, notably in IPC education and training, allocation of a protected and dedicated budget for IPC activities, and in the use of multi-modal strategies for implementing IPC programmes.

Only 26.1% of national leads reported that a protected and dedicated budget was available for IPC. These findings have important policy implications at all levels in terms of promoting IPC to improve quality care and patient safety. Functioning and effective IPC programmes require sustained financial and political support to ensure adequate human resources and to implement activities that can have an impact at the peripheral level [9,18]. This challenge particularly affects low-/lower-middle-income countries. Nevertheless, following recent widespread epidemics, such as the Ebola virus disease outbreak in West Africa and the 2012 Middle-East respiratory syndrome outbreak, there are positive examples of strong partnership and governmental investment in IPC. It has indeed been encouraging that successful IPC national programmes have been developed and implemented in Sierra Leone and Liberia due to political commitment [30,31]. Barriers in the African region are well known and related to a lack of finance and specialized IPC staffing in healthcare systems, resulting in suboptimal preparedness against emerging infectious diseases and compromising global health security.

Two-thirds of countries reported IPC national guidelines, but only one-third had an implementation strategy in place with predefined roles and responsibilities; an even lower proportion reported evaluation of compliance with guidelines. Guidelines alone are not sufficient to promote best practices [32]. In Israel, Schwaber and colleagues reported that the ability to achieve high-level compliance with national mandatory guidelines was directly correlated with the successful containment of a widespread carbapenem-resistant Enterobacterales outbreak and a reduction of HCAI rates [33]. It is critical to conceive strategies to communicate the knowledge behind evidence-based guidelines to stakeholders and to implement monitoring processes that continuously measure and provide feedback on compliance and performance.

Our findings on IPC education and training offer a mixed picture of activities, highlighting the variability across regions and income levels, and identifying gaps compared to international standards [34,35]. In-service training was reported to be the modality most frequently used to deliver IPC education; in many cases this may have been triggered by the need to prepare for, or respond to, outbreaks. However, this is a positive finding because IPC training of front-line staff is one of the WHO-recommended minimum requirements for IPC and it potentially ensures integration of IPC best practices within clinical care delivery [17]. Nevertheless, it is essential that any health professional be trained early in their educational pathway on the importance of avoiding harms due to infections acquired by patients, health workers and others during care delivery. The lower rate of undergraduate training might be related to lack of formal recognition of the importance of IPC practices across disciplines, and it requires attention by educational bodies and agreement on a common basic IPC curriculum. This could be achieved by creating standardized curricula templates and publishing guidance for the development of undergraduate IPC curricula adapted to different healthcare professions. The relatively low percentage of countries with a postgraduate IPC curriculum might be due to the lack of recognition of IPC as a discipline requiring specific specialization and a clear career pathway. WHO has highlighted the importance of adequate training of those in charge of IPC and recently defined what core competencies IPC professionals are expected to have [19,36,37]. Countries should create a curriculum and opportunities for postgraduate IPC training; some national and international courses are available (https://www.who.int/infection-prevention/about/GIPCN_Training-Courses/en/) and this need has recently been addressed by the European Society for Clinical Microbiology and Infectious Diseases, which has developed a course leading to certification for IPC specialists in the European region [38].

National systems of prospective HCAI surveillance were reported by less than half of participating countries, with major gaps in low-income countries and in the African and Eastern Mediterranean regions (3.8% and 25%, respectively). These results are similar to a previous WHO survey of 2010 reporting that only 15.7% (23/147) of developing countries had a functioning HCAI surveillance system in place [26]. These findings highlight an urgent need to put HCAI surveillance on the political agenda of many countries. National surveillance systems, coupled with timely feedback, are crucial in understanding the burden of HCAI and AMR and for early outbreak detection, and to drive action towards prevention [18,36,39]. Our survey, together with other reports, shows that the implementation of IPC and surveillance programmes varies disproportionally between high-income and low/middle-income countries [39,40]. This difference can be explained by the fact that HCAI surveillance requires resources both in staffing and microbiology capacity, including technical expertise.

Variations were observed in nationally co-ordinated monitoring of key IPC-related indicators. Having these systems in place is critical to provide actionable data to improve practices locally and helps to reduce HCAIs and AMR. In a recent longitudinal study in Australia, Grayson *et al.* demonstrated the impact of a national hand hygiene programme that ensured nationwide practices monitoring for more than a decade. A 15% reduction in the incidence of healthcare-associated *S. aureus* bacteraemia was observed for every 10% increase in hand hygiene compliance [41].

Our study revealed that 51.1% of IPC national focal points had a clear understanding of multi-modal strategies, including bundles, and 52.3% used this approach to implement IPC interventions at national level. Hand hygiene was the most common intervention for which a multi-modal strategy was applied. A recent German study also reported a lack of implementation of this core component, despite being the approach most supported by scientific evidence for its effectiveness to reduce HCAIs and AMR at the national and facility level [9,12,18,19]. The explanation of this success relies on improvement programmes that implement interventions in a holistic way and taking multiple elements into account, including human factors and implementation science [42]. While these strategies are extremely successful once implemented, they require a shift in the thinking of IPC professionals and decision-makers, and an investment of significant energy. This might explain why not all countries have adopted them consistently so far.

This global survey not only contributes to continued worldwide awareness about the importance of IPC, but also helps participants to review gaps and priorities for action and to set targets to improve national IPC programmes, thereby driving containment of AMR and prevention of HCAI including preparedness for outbreaks of highly transmissible diseases. Results from this global survey offer reference data on implementation of IPC core components and will allow future standardized comparisons across the WHO regions and World Bank income levels.

Our survey has limitations. First, we were unable to achieve the desired targeted sample size and the number of responses from the South-East Asia region was particularly low and non-representative. Second, selection bias cannot be excluded as we assessed IPC progress only in those countries that had signed the ‘Clean Care is Safer Care’ pledge to reduce HCAIs between 2005 and 2017. In addition, participating countries might have been different from non-participating countries. Third, we cannot exclude the possibility of reporting bias. Although this was not an online survey and we had the opportunity to discuss answers during in-depth interviews, we relied on self-reporting from IPC country leads. Thus, reported data may not fully reflect the objective status of implementation of IPC at the national level. Nevertheless, the confidentiality principles guiding information-sharing and data-reporting may have mitigated this risk.

In conclusion, this first global survey on the implementation of national IPC core components in 88 countries identified deficiencies across the six WHO regions and World Bank income levels.

Sustained high-level commitment is urgent and indispensable to ensure that the WHO core components for IPC, or at least their minimum requirements, are implemented to avoid patient harm within regular healthcare delivery and meet the challenges of global and local crises [17]. WHO implementation resources to support countries in adopting the recommended IPC core components at the national level are available for countries to use evidence-based approaches and adapt them locally [42]. The fight against the coronavirus disease 2019 (COVID-19) pandemic has placed infection prevention firmly in the spotlight and brought to attention the very real danger to the population worldwide if existing IPC gaps in healthcare systems are ignored. The IPC core components offer an actionable structure for benchmarking healthcare systems and for supporting countries to work towards the goal of quality universal health coverage and health security for all.
