# Supplementary material for: Implementation of the infection prevention and control core components at the national level: a global situational analysis
Source: J Hosp Infect. 2021 Feb;108:94–103. doi: 10.1016/j.jhin.2020.11.025 (PMC7884929; doi:10.1016/j.jhin.2020.11.025)
Supplement: Supplementary data [file mmc1.docx]

**Implementation of the infection prevention and control core components at the national level: a global situational analysis ~~with implications for the COVID-19 pandemic~~**

**Appendix A. Supplementary material**

**Table SI**

**WHO national infection prevention and control core components**

| **CORE COMPONENT** | **RECOMMENDATION** | **INDICATORS TO ASSESS IMPLEMENTATION** |
| --- | --- | --- |
| 1. IPC programmes | Establish an active, stand-alone IPC programme for the purpose of preventing HCAI and combating AMR through IPC good practices. | • Programme objectives, functions, and activities clearly outlined. |
|  |  | • Technical team of trained infection preventionists in place. |
|  |  | • Dedicated IPC budget allocated. |
|  |  | • Evidence that the IPC programme is linked with other relevant programmes and professional organisations. |
| 2. Evidence-based guidelines | Develop evidence-based national IPC guidelines and related implementation strategies. Ensure health-care workers’ education and training on guideline recommendations and system monitoring of adherence with guideline recommendations. | • Essential IPC guidelines developed or adapted from international standards. |
|  |  | • Necessary infrastructure and supplies to enable guideline implementation in place/being addressed. |
|  |  | • Measures to support and mandate health-care worker education and training on the guidelines in place. |
|  |  | • System to monitor adherence with guideline recommendations in place. |
| 3. Education and training | Support IPC education and training of the health workforce. | • Curricula target audience, learning objectives, competencies and teaching strategy developed. |
|  |  | • Pre-graduate curricula developed or under development. |
|  |  | • Postgraduate IPC curricula developed or under development, |
|  |  | • New employee orientation and in-service continuous training on IPC  developed or under development. |
| 4. Surveillance | Establish HCAI surveillance programmes and networks that include mechanisms for timely feedback and can be used for benchmarking purposes. | • Support and engagement by governments and authorities for IPC surveillance secured. |
|  |  | • Human and financial resources secured. |
|  |  | • Adequate microbiology and laboratory capacity and quality in place or under development- at least in national reference laboratories. |
|  |  | • Surveillance strategy developed: |
|  |  | ◦ clear objectives |
|  |  | ◦ standardised case definitions |
|  |  | ◦ methods |
|  |  | ◦ process for data analysis, reporting, and evaluation of data quality. |
|  |  | • Specific training for data collectors established. |
| 5. Multimodal strategies | Coordinate and facilitate the implementation of IPC activities through multimodal strategies adapted to the local context. | • Multimodal implementation strategies according to WHO definitions identified and actively promoted to prevent specific types of HCAIs and/or AMR. |
|  |  | • Requisite funding identified to support a multimodal approach. |
|  |  | • Evidence of IPC integration with other quality improvement/safety/accreditation programmes demonstrated. |
|  |  | • Evidence of local adaption of multimodal implementation strategies demonstrated. |
|  |  | • Evidence of monitoring compliance with the strategies and impact of the intervention on desired outcomes demonstrated. |
| 6. Monitoring, audit and feedback | Establish a monitoring and evaluation programme to assess the extent to which standards are being met and activities are being performed according to the programme's goals and objectives. Consider using hand hygiene monitoring with feedback as a key performance indicator. | • Hand hygiene monitoring with feedback established as a key performance indicator at national level. |
|  |  | • Other important IPC process indicators determined. |
|  |  | • Strategy for using the data for action developed. |
|  |  | • Regular reports produced and distributed. |
|  | | |

**Figure S1.** First Global Patient Safety Challenge, **“**Clean Care is Safer Care”: geographical distribution of WHO Member States (n=140 as of December 2017) that formally pledged their support to implement actions to reduce healthcare-associated infections.


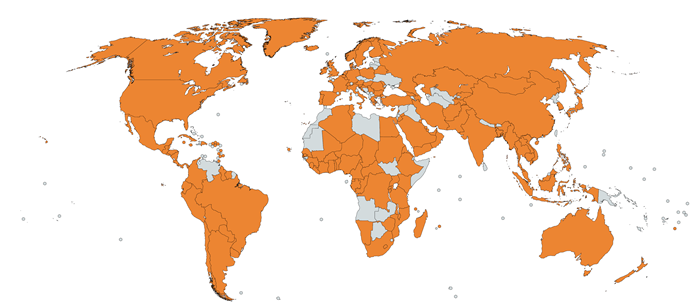


**Figure S2.** “Clean Care is Safer Care” countries* (n=88) participating in the study by WHO region**.**


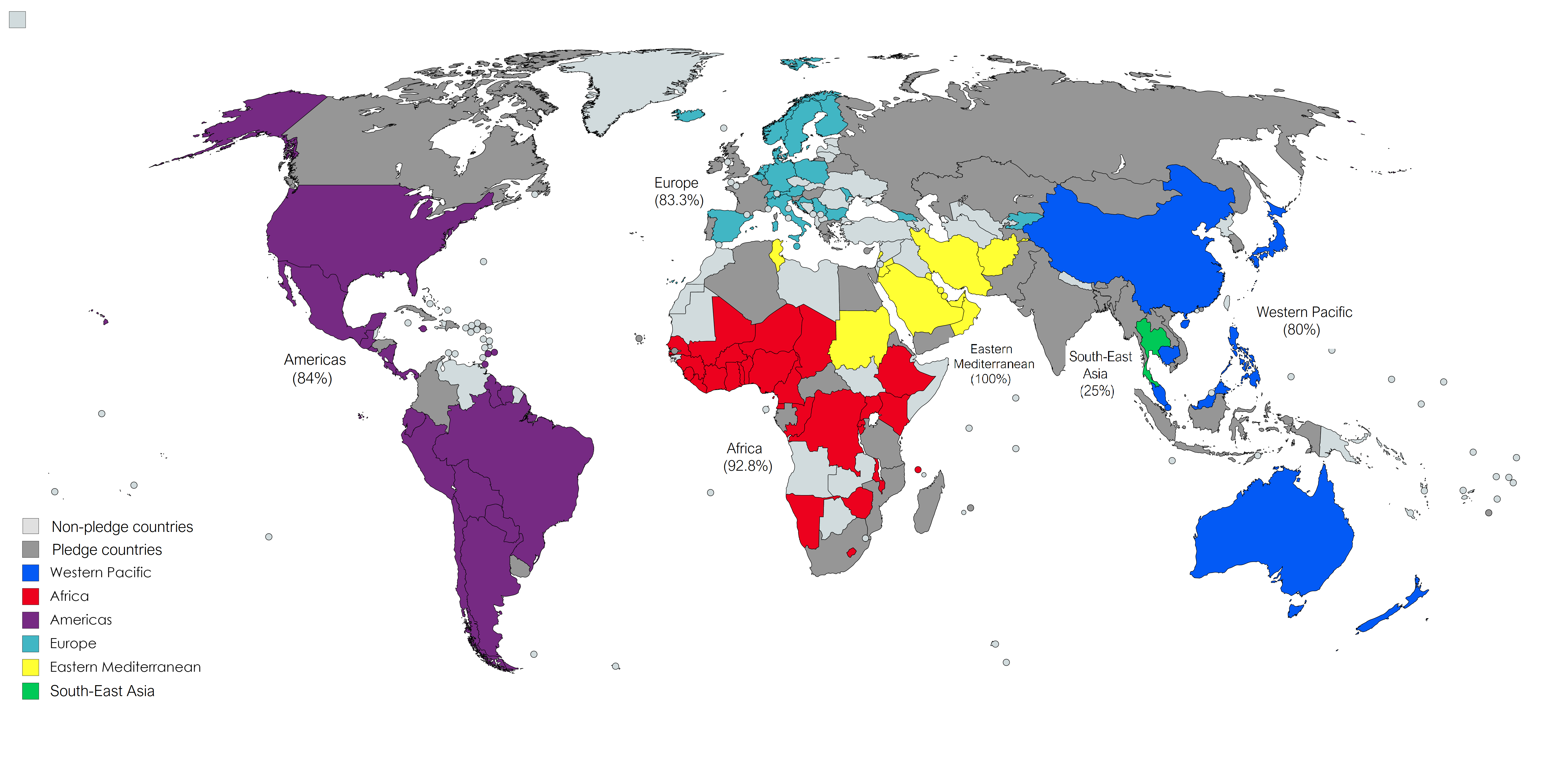


*****WHO Member States which made formal statements pledging their support to implement actions to reduce health-care-associated infections up to 2017: African region (n=26); region of the Americas (n=21); Eastern Mediterranean region (n=12); European region (n=20); South-East Asian region (n=1); Western Pacific region (n=8).

**Figure S3. “**Clean Care is Safer Care” participating countries situational analysis, 2017-2018 (n=88): results of the six core components of infection prevention and control programmes stratified by WHO region**.**

**
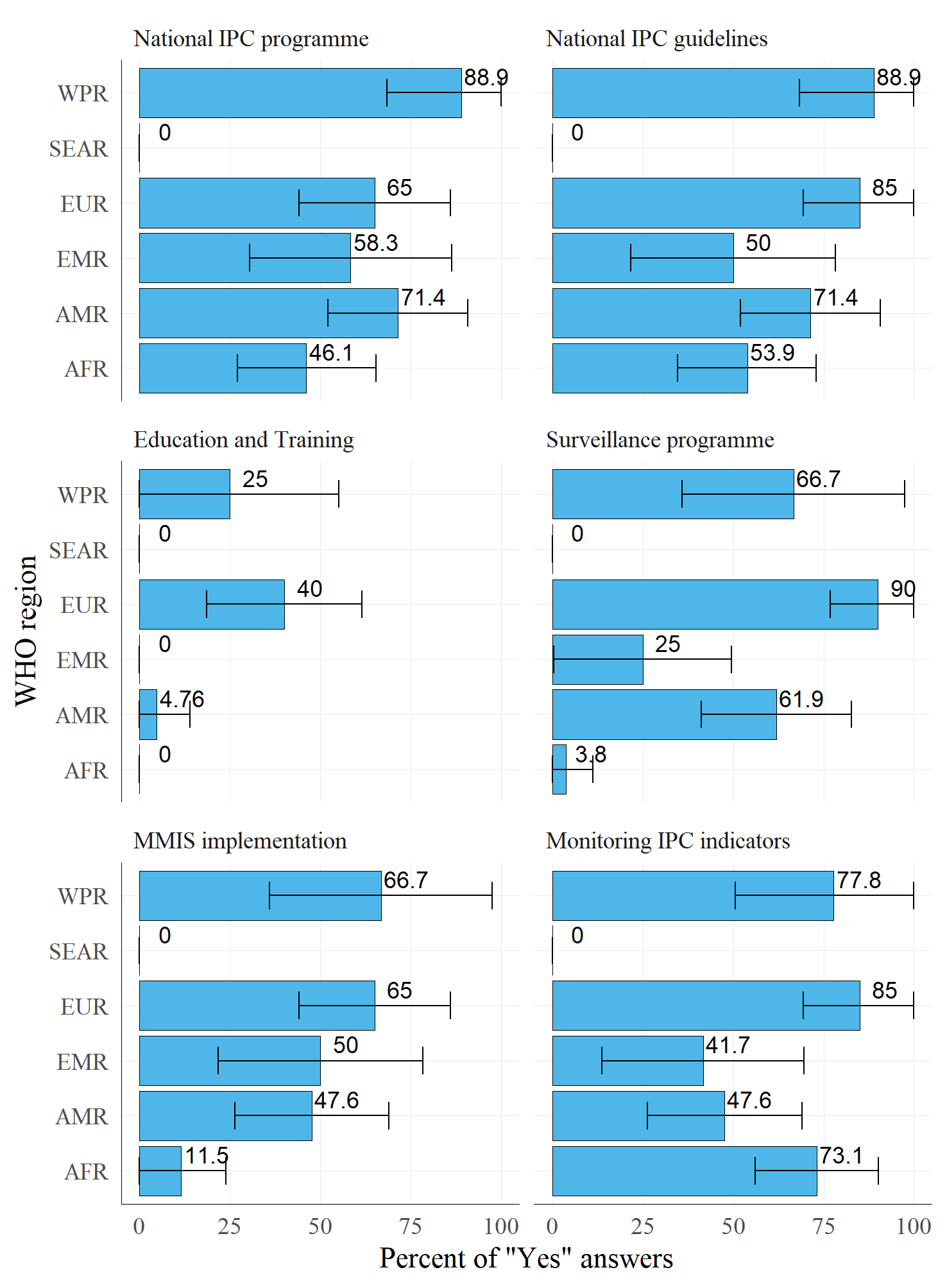
**

Footnote: Bars and whiskers indicate the overall percentage of “Yes” responses with 95% confidence intervals. African region AFR (n=26 countries); Region of the Americas AMR (n=21); Eastern Mediterranean region EMR (n=12); European region EUR (n=20); South-East Asian region SEAR (n=1); Western Pacific region WPR (n=8).

Abbreviations: IPC: infection prevention and control; MMIS: multimodal improvement strategy

**Figure S4. “**Clean Care is Safer Care” participating countries situational analysis, 2017-2018 (n=88): results of core component 3 (education and training) stratified by WHO region.

**
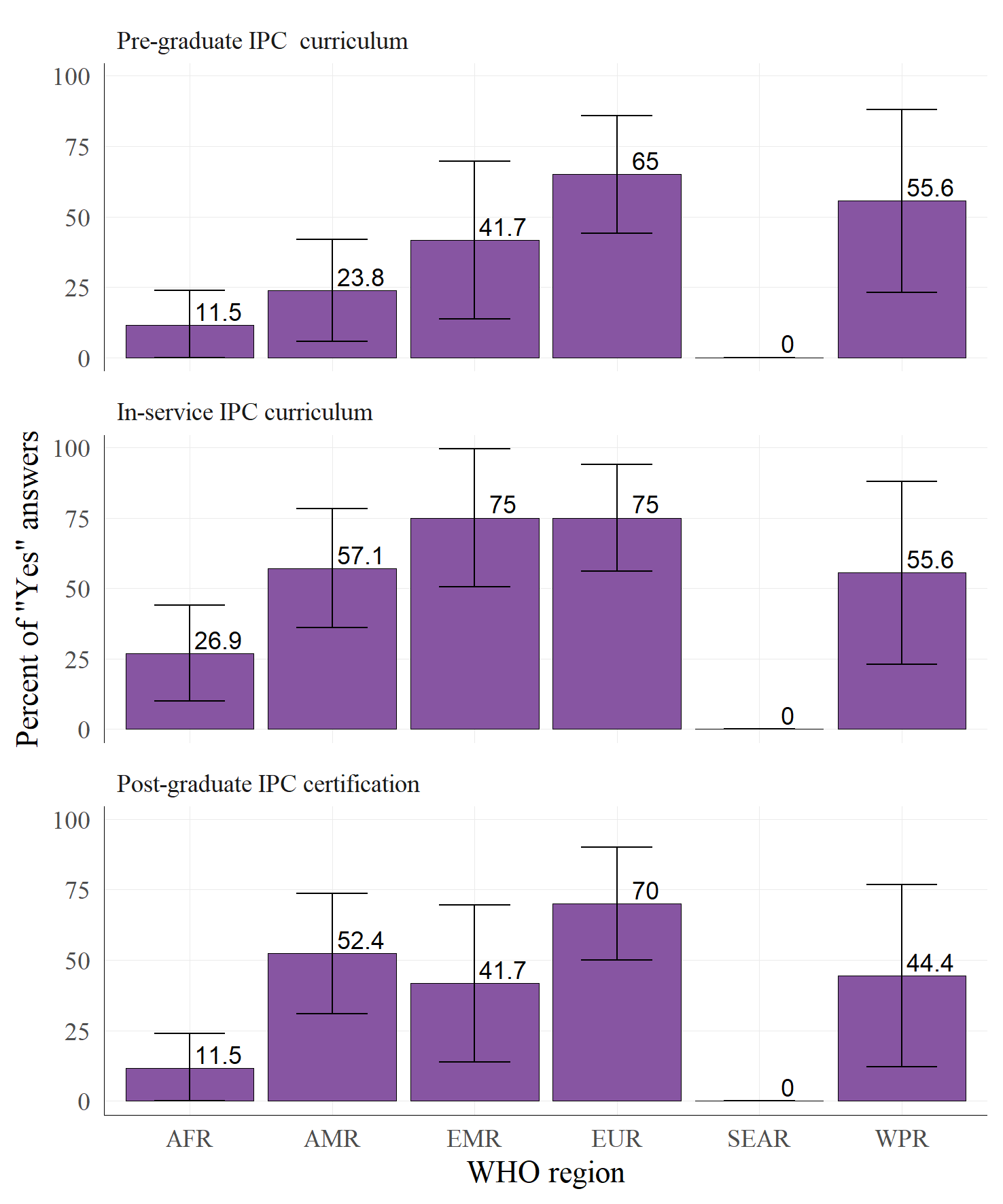
**

Footnote: Bars and whiskers indicate the overall percentage of “Yes” responses with 95% confidence intervals. African region AFR (n=26 countries); Region of the Americas AMR (n=21); Eastern Mediterranean region EMR (n=12); European region EUR (n=20); South-East Asian region SEAR (n=1); Western Pacific region WPR (n=8).

Abbreviations: IPC, infection prevention and control; HCAI, health-care-associated infection; AMR, antimicrobial resistance.

**Questionnaire used for semi-structured interiews**

1. **Infection prevention and control (IPC) programmes**
2. Is there a national IPC programme (with an appointed IPC technical team or a focal person)?

- Yes
- No *(go to section 2)*
- Don’t know
- Other related programmes (*please specify)*

1. Where is the national IPC programme located?

- Ministry of health
- National public health agency
- Other (*please specify)*

1. In which department is the national IPC programme located?

- Stand-alone IPC department/unit
- Quality management department (or quality assurance, etc.)
- Patient safety department
- Antimicrobial resistance department
- General public health/disease control department
- Other *(please specify)*

1. Does the national IPC programme have any of the following components established? Select all that apply.

- Clearly defined objectives
- Clearly defined responsibilities
- Trained team of infection preventionists (basic and advanced IPC training
- None of the above
- Don’t know

1. The national trained team of IPC preventionists (basic and advanced IPC training) includes (select all that apply):

- Medical professionals
- Nursing professionals
- Includes one or more dedicated professionals (ie, with no shared responsibilities with other departments)
- Other *(please specify)*

1. Has the IPC team a protected and dedicated budget supported by national authorities?

- Yes
- No *(go to question 8)*
- Don’t know

1. Is the dedicated IPC budget used for any of the following? Select all that apply:

- IPC programme
- IPC guidelines
- IPC education and training
- Surveillance
- Multimodal strategies
- Monitoring/audit of IPC
- Workload, staffing, and bed occupancy
- Built environment, materials and equipment at facility level
- Other *(please specify)*

1. Is IPC included in the national action plan to combat antimicrobial resistance?

- Yes
- No *(go to section 2)*
- Don’t know

1. If YES, what are the IPC core components that are explicitly mentioned in the national action plan to combat antimicrobial resistance? Select all that apply:

- IPC programme
- IPC guidelines
- IPC education and training
- Surveillance
- Multimodal strategies
- Monitoring/audit of IPC
- Workload, staffing, and bed occupancy
- Built environment, materials and equipment (eg, water, sanitation, and hygiene) at facility level
- Other *(please specify)*

**Additional notes for this section:**

1. **IPC guidelines**
2. Are there national IPC guidelines?

- Yes
- No *(go to section 3)*
- Don’t know

1. Do national IPC guidelines address any of the areas below? Select all that apply:

- IPC organisation and management
- Standard precautions
- Hand hygiene
- Transmission-based precautions
- Surgical site infection
- Central-line-associated bloodstream infection
- Catheter-associated urinary tract infection
- Hospital-acquired pneumonia/ventilator-associated pneumonia
- *Clostridioides difficile* infection
- Methicillin-resistant *Staphylococcus aureus*
- Carbapenem-resistant Enterobacterales and/or *Acinetobacter baumannii* and/or *Pseudomonas aeruginosa*
- Outbreak management
- Other *(please specify)*

1. Which of the following statements are applicable to national IPC guidelines in your country? Select all that apply:

- Guidelines have been developed from international standards
- Local and other evidence was used to develop the guidelines
- The national team organises staff education on issued guidelines
- Implementation strategy for guidelines is in place with clear roles and responsibilities
- Compliance with guidelines is evaluated by monitoring parameters with regard to process and outcome
- Don’t know

**Additional notes for this section**

**3. IPC education and training**

1. Is there a national pre-graduate IPC university curriculum in place or under development for students in the health sciences faculty?

- Yes
- No (*go to question 15)*
- Don’t know

1. Which pre-graduate courses receive IPC training?

- Medical
- Dentistry
- Nursing
- Allied health care professionals. If allied health care professionals *(please specify)*

1. Is there is an in-service IPC curriculum in the health domain?

- Yes
- No*(go to question 17)*
- Don’t know

1. Which professions are included in the in-service training?

- Medical
- Dentistry
- Nursing
- Allied health care professionals *(please specify)*
- Don’t know

1. Is there a postgraduate training certification programme for professionals to become IPC specialists?

- Yes
- No*(go to question 21)*
- Don’t know

1. Which professions are included in the certification training programme for IPC trained specialist?

- Physicians (eg, infectious disease physicians)
- Microbiologists
- Nurses
- Other laboratory-based staff
- Other *(please specify)*
- Don’t know

1. Which is the body providing certification for professionals to become IPC specialists?

- Government
- Private institutions
- Other professional bodies: (for example infectious disease/ infection prevention and control associations/ university)

1. Which international standards are used to develop programmes for IPC specialists? Select all that apply.

- WHO guidelines on core components of IPC programmes
- Other WHO guidelines *(please specify)*
- United States Centers for Disease Control and Prevention guidelines *(please specify)*
- Core competencies in IPC defined by “Training in Infection Control in Europe” (TRICE)
- Core competencies in IPC defined by the Association of Professionals in Infection Control and Epidemiology (APIC) or the Certification Board of Infection Control and Epidemiology (CBIC)
- Other local guidelines *(please specify)*
- Don’t know
- Others *(please specify)*

1. Are there Master programmes in IPC?

- Yes
- No *(go to section 4)*
- Don’t know

1. Are there doctoral (PhD) programmes in IPC?

- Yes
- No
- Don’t know

**Additional notes for this section**

1. **Surveillance**
2. Is there a national programme/system for health-care-associated infection surveillance?

- Yes
- Yes, but included in other communicable diseases surveillance programme/systems
- No *(go to section 5)*
- Don’t know

1. Do you have at least one good-quality national reference laboratory?

- Yes
- No
- Don’t know

1. What health-care-associated infections are included in the national health-care-associated infection surveillance* programme/system? Select all that apply:

**Surveillance definition: data collection and analysis to convert these data into statistics; interpretation of this analysis to produce information for dissemination to those who can take appropriate action.*

|  | No surveillance | Mandatory surveillance | Voluntary surveillance | Don’t know |
| --- | --- | --- | --- | --- |
| Catheter-associated urinary tract infections | ☐ | ☐ | ☐ | ☐ |
| Hospital-acquired pneumonia/ventilator-associated pneumonia | ☐ | ☐ | ☐ | ☐ |
| Central-line-associated bloodstream infections | ☐ | ☐ | ☐ | ☐ |
| Surgical site infections | ☐ | ☐ | ☐ | ☐ |
| *Clostridioides difficile* | ☐ | ☐ | ☐ | ☐ |
| Methicillin-resistant *Staphylococcus aureus* | ☐ | ☐ | ☐ | ☐ |
| Carbapenem-resistant Enterobacterales and/or*Acinetobacter baumannii* and/or *Pseudomonas aeruginosa* | ☐ | ☐ | ☐ | ☐ |
| Extended-spectrum beta-lactam- producing *Enterobacterales or Escherichia coli?* | ☐ | ☐ | ☐ | ☐ |
| Outbreak detection | ☐ | ☐ | ☐ | ☐ |
| Other antimicrobial resistance/susceptibility patterns | ☐ | ☐ | ☐ | ☐ |

Others (*please specify*)

**Additional notes for this section**

1. **Multimodal strategies***
2. Is there a clear understanding of the meaning of a “multimodal improvement strategy”?

- Yes,
- No
- Don’t know

1. How do you define multimodal improvement strategies?

1. Does the national IPC team support and coordinate the use of a multimodal improvement strategy to implement IPC programmes and practices at the facility level?

******* ***Multimodal improvement strategy explained:*** The use of multiple approaches that will likely influence the behaviour of the target audience to make the necessary improvements that will impact on patient outcome when used in combination.

***WHO recommends five general areas:***

1. System change (infrastructure, equipment, and other resources, eg., alcohol-based handrub at the point of care).
2. Training and education (to enhance/improve health-care workers’ competency).
3. Monitoring and feedback (to provide assurance that practice is improving and to drive change).
4. Advocacy and communications (to promote the desired actions at the right time and at the right place).
5. A culture of safety (to facilitate an organisational climate that values the intervention).

- Yes,
- No (*go to section 6)*
- Don’t know

1. Which outcomes do the multimodal strategies target? Check all that apply:

- Multimodal strategies for reducing antimicrobial resistance
- Multimodal strategies for improving hand hygiene compliance
- Multimodal strategies for reducing surgical site infections
- Multimodal strategies for reducing central-line-associated bloodstream infections
- Multimodal strategies for reducing catheter-associated urinary tract infections
- Multimodal strategies for reducing hospital-acquired pneumonia/ventilator-associated pneumonia
- Other (*please specify*)

**Additional notes for this section**

1. **Monitoring/audit of IPC practices and feedback**
2. Are IPC-related indicators* included in the national monitoring and evaluation efforts?

*A performance indicator is a type of performance measurement that evaluates periodically the progress and achievement of operational and strategic goals

- Yes
- No *(go to question 33)*
- Don’t know

1. Which indicators relevant to IPC are regularly (at least annually) monitored? Check all that apply:

- Hand hygiene compliance
- Alcohol-based handrub consumption
- Water, sanitation, and hygiene
- Antibiotic consumption
- Health-care worker staffing levels (eg, health-care worker-to-patient ratio)
- Bed occupancy
- Other (*please specify)*

1. Who reports to national authorities about outcome and process indicators in IPC? Check all that apply:

- Individual acute care hospitals directly
- Individual long-term care facilities directly
- Regional health departments
- Other surveillance networks *(please specify)*
- Other *(please specify)*

1. **Demographics of the respondent**

Country:

Name:

Professional position

Years of experience in this position:

E-mail address:

Telephone number:

**Additional notes for this section**
